# Early Staphylococcal Periprosthetic Joint Infection (PJI) Treated with Debridement, Antibiotics, and Implant Retention (DAIR): Inferior Outcomes in Patients with Staphylococci Resistant to Rifampicin

**DOI:** 10.3390/antibiotics12111589

**Published:** 2023-11-03

**Authors:** Hannah K. Eriksson, Stergios Lazarinis, Josef D. Järhult, Nils P. Hailer

**Affiliations:** 1 Department of Surgical Sciences, Section of Orthopaedics, Uppsala University, 751 83 Uppsala, Sweden; lazarinis.stergios@uu.se (S.L.); nils.hailer@uu.se (N.P.H.); 2 Zoonosis Science Center, Department of Medical Sciences, Uppsala University, 751 83 Uppsala, Sweden; josef.jarhult@medsci.uu.se

**Keywords:** periprosthetic joint infection, PJI, debridement, antibiotics, implant retention, DAIR, rifampicin, staphylococcus

## Abstract

It is unknown how rifampicin resistance in staphylococci causing a periprosthetic joint infection (PJI) affects outcomes after debridement, antibiotics, and implant retention (DAIR). We thus aimed to compare the risk of relapse in DAIR-treated early PJI caused by staphylococci with or without rifampicin resistance. In total, 81 patients affected by early PJI were included, and all patients were treated surgically with DAIR. This was repeated if needed. The endpoint of relapse-free survival was estimated using the Kaplan–Meier method, and Cox regression models were fitted to assess the risk of infection relapse for patients infected with rifampicin-resistant bacteria, adjusted for age, sex, type of joint, and type of index surgery. In patients with rifampicin-resistant staphylococci, relapse was seen in 80% after one DAIR procedure and in 70% after two DAIR procedures. In patients with rifampicin-sensitive bacteria, 51% had an infection relapse after one DAIR procedure and 33% had an infection relapse after two DAIR procedures. Patients with rifampicin-resistant staphylococcal PJI thus had an increased adjusted risk of infection relapse of 1.9 (95% CI: 1.1–3.6, *p* = 0.04) after one DAIR procedure compared to patients with rifampicin-sensitive bacteria and a 4.1-fold (95% CI: 1.2–14.1, *p* = 0.03) increase in risk of infection relapse after two DAIR procedures. Staphylococcal resistance to rifampicin is associated with inferior outcomes after DAIR. These findings suggest that DAIR may not be a useful strategy in early PJI caused by rifampicin-resistant staphylococci.

## 1. Introduction

Periprosthetic joint infection (PJI) after total joint arthroplasty (TJA) is a devastating complication associated with increased morbidity and mortality [1,2,3,4]. PJI is predominantly an early complication and is the second most frequent indication for a revision surgery within 2 years after an index surgery [5]. Treatment of PJI often requires multiple surgical interventions and long-term antibiotic administration [6,7]. Staphylococcus aureus (*S. aureus*) and coagulase-negative staphylococci (CoNS) are the most frequent causative bacteria [8,9] and contribute to up to 60% of PJIs in hips and knees [10,11,12,13,14,15,16]. The proportions of PJIs caused by *S. aureus* and CoNS appear to be relatively similar, but PJIs caused by these two types of bacteria vary in the time until detection. Highly virulent microorganisms such as *S. aureus* generally cause early infections more often (~35% of cases) [16,17], whereas the less virulent CoNS are more frequent in delayed PJI.

Both *S. aureus* and CoNS are typically found as commensals on human skin and mucous membranes. They have the capability to adhere to prosthetic implants and form biofilms [18,19]. A biofilm is a complex and well-structured aggregation of a single species or multiple species of microorganisms embedded in a matrix. Mature biofilms develop within 2 to 6 weeks after inoculation [20,21], although the process of biofilm formation varies greatly between bacterial species [22,23]. Biofilm-associated infection is one of the most common causes of orthopedic implant failure [24] since biofilm adheres to the implant and generally cannot be eliminated solely using antimicrobial agents. Biofilm must be removed, and surgical treatment entails either debridement, antibiotics, and implant retention (DAIR) or a one- or two-stage revision of the implant [25]. In cases of early PJI, the recommended surgical strategy is the DAIR procedure, which does not require the removal of cemented or osseointegrated uncemented prostheses [26]. 

DAIR involves a comprehensive synovectomy, eliminating all inflamed tissue, clots, and fibrinous material, followed by a pulsatile joint lavage. When present, modular components such as femoral heads or polyethylene liners are exchanged [27]. This surgical method demonstrates varying rates of infection control, ranging from 12 to 80% [28,29,30,31,32]. The long-term success of a DAIR procedure is dependent on numerous factors, including the duration of symptoms before revision surgery [28] and a range of other factors that may contribute to worse treatment outcomes (e.g., the age of the implant, failure to perform an exchange of modular components, the presence of a sinus tract, and manifest implant loosening) [33,34,35,36].

Antibiotics are systemically administered following all surgical interventions for PJIs, including DAIR. In most cases, the treatment starts with broad-spectrum antibiotics given intravenously for an initial period. After pathogens are identified and antimicrobial susceptibility is defined, antimicrobial therapy can be individually tailored [35,36,37,38,39,40]. Following a DAIR procedure, it is recommended to initiate an intravenous antibiotic treatment without any biofilm-active component in the initial post-surgery weeks. The choice of antibiotics is adjusted according to the specific bacteria responsible for the infection, and doses are adjusted based on the patient’s size and kidney function. In cases involving *S. aureus* and CoNS, a common choice is a β-lactam or glycopeptide intravenous antibiotic [41,42], followed by an oral administration regimen. This oral phase may include rifampicin combined with fluoroquinolone, fusidic acid, or clindamycin, depending on individual bacterial resistance patterns and patient requirements [43,44,45,46,47]. Since biofilm develops on implants during staphylococcal PJI and since implants are not removed during DAIR procedures, rifampicin, as the only antibiotic agent with the proven ability to penetrate biofilm, is very frequently used after DAIR procedures. This is, of course, dependent on the resistance profiles of the causative bacteria and is preferred due to its remarkable effectiveness against microorganisms with biofilm-forming capabilities [25,39,48,49,50]. Rifampicin acts via the inhibition of DNA (deoxyribonucleic)-dependent RNA polymerase, leading to a suppression of RNA synthesis and cell death [51]. Due to the substantial risk of developing antibiotic resistance, the use of rifampicin as a monotherapy for the treatment of PJI is not encouraged [52]. 

In a recent study, a comparative analysis was carried out to assess treatment outcomes in patients with PJI caused by bacteria that were either resistant or sensitive to rifampicin. The results indicated notably inferior treatment results among individuals affected by rifampicin-resistant strains [53]. In a study examining the genomic variations and virulence-associated characteristics in 111 staphylococcal strains, an association between the presence of a strong biofilm-forming ability and antibiotic resistance was found in *S. aureus*. This association was linked to a higher likelihood of treatment failure in cases of PJI [54]. However, the impact of rifampicin resistance on treatment outcomes in cases of early PJI managed with DAIR is unknown. We therefore aimed to investigate whether resistance to rifampicin affects the risk of infection relapse after DAIR in early PJI caused by staphylococci (*S. aureus* or CoNS). 

## 2. Results

Within our cohort comprising a total of 81 patients, there was a nearly even distribution between genders, with 42 of the patients (52%) being women. The average age of the patients was 72 years, spanning a range from 41 to 93 years. 

Within the study cohort, 30 patients had a PJI in a knee arthroplasty and 51 had a PJI in a hip arthroplasty. The cohort was followed up for an average duration of eight years, with individual follow-up periods ranging from a minimum of 2 years to a maximum of 18 years. PJI manifested after the primary surgery in the majority of patients (n = 63; 78% of all cases), while 18 patients (22%) experienced a PJI following a revision surgery. Of all PJIs, 64 cases were attributed to CoNS and 17 cases were attributed to *S. aureus*. In total, 20 (25%) bacterial strains were resistant to rifampicin, all of which were CoNS, whereas all *S. aureus* strains were sensitive to rifampicin. 

The patients exposed to rifampicin-resistant staphylococci included 12 women and 8 men. This rifampicin-resistant group consisted of 4 cases involving hip arthroplasty and 16 cases with knee arthroplasty. In contrast, the rifampicin-sensitive group encompassed 30 women and 31 men, involving 25 hip arthroplasties and 36 knee arthroplasties. The mean time interval between the index surgical procedure and the initial DAIR procedure was 24 days, ranging from a minimum of 7 days to a maximum of 42 days.

### 2.1. Treatment Outcome after One DAIR Procedure

In the entirety of our cohort, 47 patients, constituting approximately 58% of the total cohort, had an infection relapse after one DAIR procedure. In the subset of patients with rifampicin-resistant strains (exclusively affected by CoNS), 16 out of 20 (80%) suffered from an infection relapse. This contrasted with the rifampicin-sensitive group, where 31 out of 61 individuals (51%) experienced an infection relapse. 

**Figure 1:**
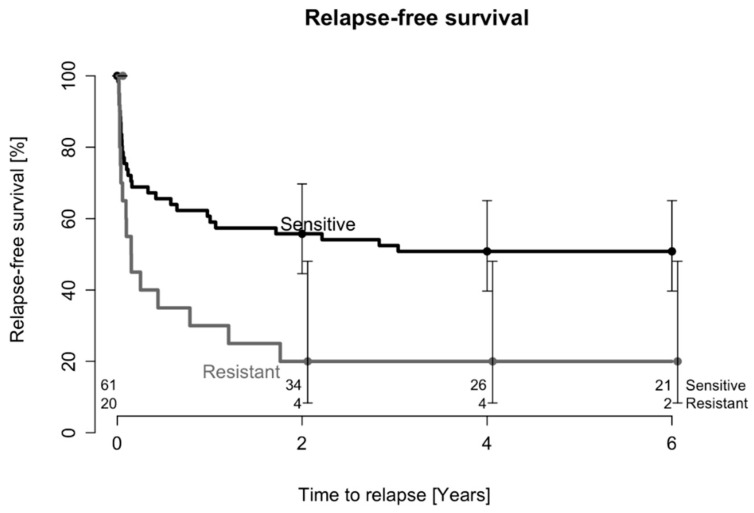
Probability of relapse after one DAIR procedure in patients affected by rifampicin-resistant or rifampicin-sensitive staphylococci. The numbers of patients at risk during the follow-up period are indicated above the x-axis.

Kaplan–Meier estimates of relapse-free survival after one DAIR procedure are presented in Figure 1, and the relapse-free survival was statistically significantly inferior in those infected by rifampicin-resistant bacteria (*p* = 0.02). Using Cox regression modeling, we obtained adjusted hazard ratios (HRs) for the risk of infection relapse and found a risk of 1.9 (95% CI: 1.1–3.6, *p* = 0.04) in patients in which the causative staphylococci were resistant to rifampicin. Moreover, the number of surgical interventions (another DAIR procedure, a one- or two-stage revision, or the permanent removal of the implant) required to achieve successful treatment after the first DAIR procedure was statistically significantly higher in the group of patients with rifampicin-resistant bacteria (mean: 2.8; SD: 1.7; range: 1–7) than in those with rifampicin-sensitive agents (mean: 1.8; SD: 1.0; range: 1–5; *p* = 0.003). 

### 2.2. Treatment Outcome after Two DAIR Procedures

A relapse of the infection was observed in 17 of the 30 patients who underwent a second DAIR procedure. In the rifampicin-resistant group in which the infection relapsed after one DAIR procedure, another two patients reached successful treatment after a second DAIR procedure. In the rifampicin-sensitive group, another 11 patients achieved successful treatment outcomes after a second DAIR procedure. Thus, the relapse rates after two DAIR procedures decreased to 70% (14 of 20 patients) in the rifampicin-resistant group and 33% (20 of 61 patients) in the rifampicin-sensitive group. 

**Figure 2:**
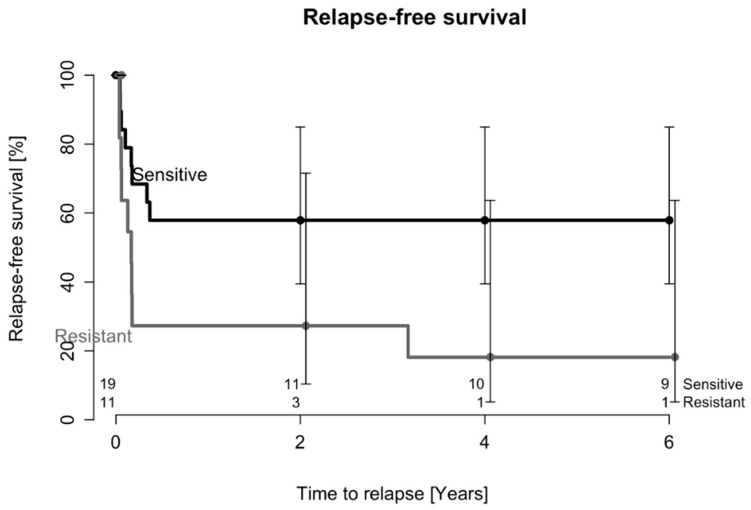
Probability of relapse after two DAIR procedures in patients affected by rifampicin-resistant or rifampicin-sensitive *staphylococci*. The numbers of patients at risk during the follow-up period are indicated above the x-axis.

The average duration between the index surgery and the second DAIR surgery was 137 days, spanning from 17 days to a maximum of 900 days. Kaplan–Meier estimates of relapse-free survival after two DAIR procedures are depicted in Figure 2, and patients affected by rifampicin-resistant staphylococci had statistically significantly inferior relapse-free survival after two DAIR procedures than patients with rifampicin-sensitive bacteria (*p* = 0.03). The adjusted HR for the risk of infection relapse after two DAIR procedures was 4.1 (95% CI: 1.2–14.1, *p* = 0.03) in patients where the causative agent was resistant to rifampicin. 

### 2.3. Analysis of PJI Caused by CoNS 

CoNS differ considerably from *S. aureus*, both in their microbiological properties and in their clinical presentation. This, together with the fact that all rifampicin-resistant strains were found among the subgroup of patients with PJI caused by CoNS, prompted us to perform a subgroup analysis of only those patients infected by CoNS, comprising a total of 64 patients. Of these, 44 had CoNS strains resistant to rifampicin. In this subgroup of 64 patients infected by CoNS, 39 patients, accounting for approximately 61% of the subgroup, had an infection relapse after one DAIR procedure. In the cohort of patients with rifampicin-resistant strains, 16 of 20 individuals (80%) had an infection relapse. In contrast, in patients with rifampicin-sensitive CoNS, a relapse was seen in only 23 of 44 patients (53%).

**Figure 3:**
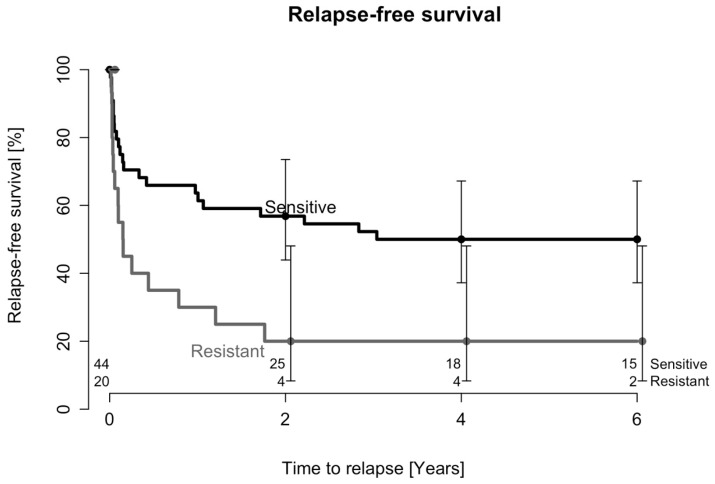
Probability of relapse after one DAIR procedure in patients affected by rifampicin-resistant or rifampicin-sensitive staphylococci in CoNS cohort. The numbers of patients at risk during the follow-up period are indicated above the x-axis.

Kaplan–Meier estimates of relapse-free survival after one DAIR procedure in patients affected by CoNS are presented in Figure 3, and the difference in relapse-free survival was statistically significant between groups (*p* = 0.011). The adjusted HR for infection relapse was 2.2 (95% CI: 1.1–4.2, *p* = 0.02) if the causative CoNS were resistant to rifampicin. Moreover, the number of surgical interventions (another DAIR procedure, a one- or two-stage revision, or the permanent removal of the implant) required to achieve successful treatment after the first DAIR procedure was significantly higher in the rifampicin-resistant group (mean: 2.6; SD: 1.7; range: 1–7) than in the rifampicin-sensitive group (mean: 1.8; SD: 1.1; range: 1–5; *p* = 0.03). 

## 3. Discussion

This study focused on the risk of infection relapse in patients with early PJI caused by *Staphylococcus* spp. with resistance to rifampicin, the most potent biofilm-active antimicrobial agent in the context of PJI. We limited our analysis to patients treated with a maximum of two DAIR interventions since further DAIR interventions after two DAIR procedures are often considered meaningless. 

The management of early PJI with DAIR is known to yield varying rates of success, ranging from 31% to 82% [33,34,35], which is understandable given the complexity of the treatment and the diversity in the baseline data. Differences in success rates may be due to the time between the index surgery and the DAIR procedure, the type of causative agent and its patterns of resistance, and the number of DAIR procedures required to achieve a successful treatment [33,34,35,55]. 

The rate of antibiotic-resistant staphylococcal strains in PJI is described to be on the ascent [56,57,58,59]. In many settings, 60 to 100% of clinical staphylococci isolates are resistant to methicillin [57,58,59], but only 55% of the bacteria in our cohort were resistant to this agent. Staphylococci can develop and act as a reservoir for genes coding for resistance mechanisms, lowering the efficiency of some antibiotics, such as rifampicin. Rifampicin is an antibiotic that is readily absorbed when taken orally, and its bactericidal effects are achieved through the inhibition of bacterial RNA polymerase. Rifampicin resistance develops through a single-step mutation in the rpoB gene encoding the β-subunit of bacterial DNA-dependent RNA polymerase [60,61,62,63]. Resistance genes can be transmitted via horizontal gene transfer [64] to other pathogens [59,65,66]. Rifampicin is widely recognized for its remarkable biofilm-penetrating capabilities, even when given orally, making it a cornerstone in the treatment of PJI. Recent research has provided insights into the role of rifampicin in PJI treatment. In cases involving patients with rifampicin-sensitive bacteria, those who received rifampicin had a lower risk of infection relapse, with 2-year infection-free survival rates of 92% compared to 83% [67]. These findings are in line with the conclusions presented by Karlsen and colleagues [68] for a randomized controlled trial comprising 48 patients who underwent DAIR for acute staphylococcal PJI. They also resonate with the results of a previous randomized study involving 48 patients who underwent DAIR for acute staphylococcal PJI. That study demonstrated that the addition of rifampicin to the standard antibiotic regimen provided a substantial benefit [34].

Given the pivotal role of rifampicin in PJI treatment and the concerning rise in rifampicin resistance in recent decades, it has become imperative to address this issue [69,70]. A study outlining the occurrence of rifampicin-resistant bacteria isolated from PJI patients reported a consistent 24% level of rifampicin resistance in PJI-associated bacteria over a 6-year period [71]. In contrast, a more recent study spanning two decades at our unit unveiled a declining trend in PJIs caused by rifampicin-resistant bacteria when comparing the time frames of 2011–2015 and 2016–2020 [67].

Rifampicin resistance can develop rapidly, particularly when rifampicin is used as a monotherapy or in the presence of a high bacterial load. This phenomenon has been observed in both *S. aureus* and CoNS isolates. Hence, it is advisable to employ rifampicin in combination with other antibiotics for the treatment of PJI. Furthermore, the administration of rifampicin should be delayed until a few days after PJI surgery, allowing the surgical revision and the initial antibiotic treatment to reduce the total bacterial load [72,73]. 

Therefore, in this study, nearly every patient initially received intravenous vancomycin during the early postoperative period, followed by various combinations of antibiotics after the wound had dried up and the resistance patterns of the causative agents had been characterized. For the treatment of rifampicin-sensitive agents, rifampicin was usually combined with ciprofloxacin or clindamycin. In the case of resistance to rifampicin, other agents such as fusidic acid, daptomycin, and linezolid were utilized.

In a study focusing on early PJI, defined as occurring within 30 days of the index surgery, patients underwent a single DAIR procedure [26], and in that study *S. aureus,* in general, conferred outcomes comparable to other microorganisms. Nonetheless, in instances of multidrug resistance, such as in the case of multiresistant *S. aureus*, the study found that only 27% of patients achieved a successful treatment outcome after DAIR when compared to cases involving more sensitive strains [26]. This was further highlighted in a study where researchers investigated the relationship between the rifampicin resistance of the bacteria responsible for PJI and the overall treatment outcomes. The authors found that the risk of infection relapse was 4.2 times higher for patients with an infection caused by rifampicin-resistant bacteria compared to those who suffered from PJIs caused by rifampicin-sensitive strains [67].

Our study reveals compelling insights, emphasizing an association between the resistance of *staphylococci* to rifampicin and the less favorable outcomes observed in the management of early PJI with DAIR procedures. These findings underscore the significant impact of rifampicin resistance on the treatment efficacy of DAIR in early PJI, emphasizing the need for a deeper understanding of this relationship to enhance patient outcomes in such scenarios. Our results align with a prior study conducted by Holmberg et al., which also reported inferior outcomes associated with rifampicin resistance. Holmberg’s study, involving 145 primary knee prostheses in need of revision, concluded that staphylococcal PJIs treated with a combination of antibiotics including rifampicin had a lower failure rate when compared to cases where rifampicin was not included in the treatment regimen [31]. Findings consistent with our study were also presented in a study that examined the influence of rifampicin resistance on treatment outcomes in patients undergoing a two-stage revision for PJI. In this study, the authors identified an increased rate of infection relapse in the rifampicin-resistant group as opposed to the rifampicin-sensitive group [53]. 

In our study, we noticed that patients with infections caused by rifampicin-resistant bacteria needed more surgical interventions to achieve a successful treatment outcome compared to individuals with infections caused by rifampicin-sensitive strains. This highlights the challenging nature of managing PJIs associated with rifampicin-resistant strains, necessitating a more comprehensive and prolonged treatment approach that may involve multiple surgical procedures. Our Kaplan–Meier estimates, used to evaluate infection-free survival following one or two DAIR procedures, indicated that when the treatment regimen for PJI is initiated within the first 6 weeks after the index procedure, any instances of treatment failure tend to manifest during the initial weeks post-surgery. This observation emphasizes the importance of monitoring patients postoperatively and taking prompt action when needed to improve the treatment outcome.

Within our study cohort, all *S. aureus* strains exhibited sensitivity to rifampicin. However, when we conducted a more specific subgroup analysis concentrating on cases of PJI attributed solely to CoNS, we observed a noteworthy pattern. In this subset, a larger proportion of the causative agents displayed resistance to rifampicin. This discrepancy led to noticeable differences in relapse-free survival between patients exposed to rifampicin-resistant CoNS and those with rifampicin-sensitive strains. These differences achieved statistical significance. This subgroup analysis strengthens the robustness of our findings, suggesting that the association between rifampicin resistance and treatment outcomes is consistent when investigating a subgroup of patients only affected by CoNS. 

This study is not without its limitations, which should be acknowledged. One such limitation is the relatively long interval between the index procedure and the first DAIR procedure. It is important to note that PJI is traditionally categorized based on the time elapsed since the initial surgery. The initial categories defined early PJI, occurring within 3 months after surgery; delayed PJI, manifesting between 3 and 24 months post-surgery; and late PJI, occurring more than two years after a surgical procedure [8,41]. In accordance with a more contemporary classification system, PJIs are categorized differently. According to this updated classification, early PJIs are defined as infections that become apparent within the initial month following a surgical procedure. Delayed PJIs, on the other hand, are diagnosed between the third and tenth week after a surgery. Late PJIs are those that occur more than ten weeks following implantation [74]. When the duration between the initial surgical procedure and the first DAIR intervention is extended, it has been associated with an increased risk of failure. Biofilm formation is initiated immediately after inoculation, but the development of biofilm in PJI typically follows a distinct pattern of maturation. For a DAIR procedure to be successful, biofilm maturation must not have progressed to the mature stage of irreversible adhesion. In addition, rifampicin-resistant bacteria can exhibit an enhanced capacity for biofilm formation. This resistance mechanism may gradually develop over time, making treatment more challenging. In other words, the more time that elapses between the primary surgery and the first DAIR procedure, the higher the risk of infection relapse. This suggests that timing plays a crucial role in the effectiveness of DAIR as a treatment for PJI [26]. A study investigating the timing of the DAIR procedure in relation to its proximity to the index surgery found that when the DAIR procedure was performed within 2–4 weeks after the index surgery, it had a HR for failure of 1.7 (with a 95% confidence interval of 1.1–2.7). In contrast, when the DAIR procedure was conducted at least 4 weeks after the index surgery, a higher HR of 2.3 was reported (with a 95% confidence interval of 1.4–3.9) [75]. Our own relapse rates have to be considered relatively high, indicating that our DAIR procedures were performed at a stage where biofilm maturation had progressed too far, and in our current practice we tend to discard DAIR as a viable treatment option once four weeks have passed after the index procedure.

Another limitation of this study is that in cases requiring two surgical interventions with DAIR, the average duration between the first DAIR surgery and the subsequent second DAIR surgery was 137 days (range: 17–900). This wide range suggests significant variability in the time intervals between the index surgery and the decision to undergo a second DAIR procedure. Some patients received the second DAIR surgery relatively soon after their initial surgery, while others experienced a much longer delay before the second procedure was performed. This variability in timing may have been influenced by individual patient factors, clinical considerations, and the evolution of PJI treatment strategies over time.

Another drawback of our study is its limited sample size. The retrospective, observational design of our study also makes it impossible to draw any further conclusions regarding why or how rifampicin resistance affects treatment outcomes, even if this knowledge would have immense value in clinical practice. Data regarding bone loss and tissue quality are also missing. 

Like all retrospective studies, our research is subject to certain inherent limitations that must be considered. One of the main concerns in retrospective studies is the possibility of selection bias. Furthermore, another limitation inherent to retrospective studies is the potential for inaccuracies or misinterpretations in the information extracted from medical records. In our study, we relied on historical medical records, and despite our best efforts to ensure data accuracy, errors or omissions may have occurred. Inaccurate or misinterpreted data could have affected the validity and reliability of our study’s results.

Considering our study’s findings, which highlight the challenges associated with rifampicin resistance, including higher relapse rates and an increased need for multiple surgical interventions to achieve a positive outcome, they prompt us to question the suitability of DAIR as the primary treatment choice when the causative *staphylococci* strains are resistant to rifampicin. It should be a core principle to minimize surgical procedures. This principle is rooted in the desire to keep surgical trauma and its associated complications to a minimum, ultimately prioritizing the overall well-being of patients. 

Our study emphasizes the importance of further research into the impact of rifampicin resistance among causative bacteria and its implications for selecting the most effective surgical intervention. Are there alternative surgical strategies that might offer more favorable treatment outcomes than DAIR for patients with early PJI caused by rifampicin-resistant staphylococci? Complete revision procedures involving one- or two-stage exchanges may provide more successful outcomes and have to be considered, even in early PJI, if it the causative agents are resistant to rifampicin. 

## 4. Patients and Methods

This retrospective study retrieved patients from our local arthroplasty register, which included information on 571 PJIs in the hip or knee. For characteristics, see Table 1. The study encompassed patients who underwent treatment for PJI between 2004 and 2013. This study included all patients treated with a DAIR procedure as the first surgical intervention for an early PJI (confirmed by at least two positive tissue cultures incubated for 14 days according to the standard procedure) with the causative bacteria identified as staphylococci spp. and data available on rifampicin susceptibility. Early PJI was defined as an infection diagnosed within 6 weeks after an index surgical procedure, where index procedures could be primary or revision surgeries of either the hip or knee. The diagnosis of PJI was based on the 2018 Musculoskeletal Infection Society (MSIS) criteria [76].

The CoNS included in this study were considered as one group and were not divided into different species.

Infection relapse was defined as the need for any further surgical intervention related to the index PJI (i.e., a repeated DAIR, subsequent one- or two-stage procedures, implant removal, or amputation), PJI-related death, or the need for a long-term suppressive antimicrobial treatment because of clinical signs of persistent PJI.

Postoperative antibiotics were administered according to the resistance pattern after a team discussion among the treating orthopedic surgeons and infectious disease specialists. The identification of CoNS and *S. aureus* was performed using commercial biotype identification kits, such as API Staph Idents. Antibiotic susceptibility testing was performed using the disc diffusion method. It is worth noting that the breakpoints utilized for microbiology analyses in routine clinical practice experienced minor changes during the study period. Until 2010, the SRGA (the Swedish Reference Group for Antibiotics) guidelines were followed [77]. For staphylococci and rifampicin, ‘sensitive’ was defined as >25 mm and ‘resistant’ was defined as <22 mm. Starting in 2011, the breakpoints were adjusted in accordance with the recommendations from NordicAST (Nordic Committee on Antimicrobial Susceptibility Testing) [78]. For staphylococci and rifampicin, ‘sensitive’ was redefined as ≥26 mm, while ‘resistant’ was redefined as <23 mm.

### Statistics

Descriptive statistics with means and ranges were used to summarize and report patient demographics. Relapse-free implant survival was estimated using a Kaplan–Meier survival analysis, and differences between groups were tested for statistical significance using a Mantel–Haenszel log-rank test. Cox regression models were fitted to estimate the risk of infection relapse, expressed as hazard ratios (HRs) with 95% confidence intervals (CIs), depending on whether a PJI was caused by rifampicin-resistant or rifampicin-sensitive staphylococci. We adjusted for the following potential confounders: sex, joint (hip or knee), type of index surgery (primary or revision), and age (dichotomized into ˂70 or ≥70 years). A chi-square test was used to compare the expected and observed numbers of surgeries in the rifampicin-resistant and rifampicin-sensitive groups. *p*-values <0.05 were considered significant. All statistical analyses were performed using SPSS version 27.0 (SPSS Inc., Chicago, IL, USA), and graphs were plotted using R software (version 4.2.1) using, among others, the package “rms”.

## 5. Conclusions

Staphylococcal resistance to rifampicin is associated with inferior outcomes in early PJI treated with DAIR procedures. In our study cohort, all rifampicin-resistant causative agents were CoNS, whereas all *S. aureus* were sensitive to this antibiotic. These findings suggest a change in practice for patients affected by PJI caused by rifampicin-resistant CoNS, as DAIR may not be a useful strategy under these circumstances. 

## Figures and Tables

**Table 1:** Characteristics of the study population.

	Rifampicin-Sensitive	Rifampicin-Resistant
Variable		
**Sex**		
Female (n = 42)	30	12
Male (n = 39)	31	8
**Age**		
˂70 (n = 34)	26	8
≥70 (n = 47)	35	12
**Joint**		
Hip (n = 51)	35	16
Knee (n = 30)	26	4
**Index surgery**		
Primary (n = 63)	49	14
Revision (n = 18)	12	6
***Staphylococcus* spp.**		
Coagulase-negative staphylococcus (n = 64)	44	20
Staphylococcus aureus (n = 17)	17	0
Duration from index surgery to first DAIR procedure (mean days (range))	24 (7–42)	25 (8–42)

## Data Availability

Data are available upon reasonable request.
